# Epidemiological characteristics and influencing factors of hand, foot and mouth disease reinfection cases in a large district of southern China

**DOI:** 10.3389/fpubh.2026.1773908

**Published:** 2026-04-01

**Authors:** Meng Ren, Azhu Han, Jinfeng Liu, Chao Wang, Xindong Zhang, Yixiong Chen

**Affiliations:** 1 Bao'an Center for Disease Control and Prevention, Shenzhen, China; 2 Shenzhen Field Epidemiology Training Project, Shenzhen, China; 3 Ministry of Education-Shanghai Key Laboratory of Children's Environmental Health, Xinhua Hospital, Shanghai Jiao Tong University School of Medicine, Shanghai, China

**Keywords:** epidemiological characteristics, hand, foot and mouth disease, influencing factors, reinfection, spatial autocorrelation

## Abstract

**Objective:**

To analyze the epidemiological characteristics of hand, foot and mouth disease (HFMD) reinfection cases in southern China and systematically evaluate key influencing factors from individual characteristics, epidemic cycles, and regional features, aiming to provide evidence for targeted prevention and control strategies.

**Methods:**

We extracted HFMD cases in a district of southern China from the China Information System for Disease Control and Prevention (2008–2024). Descriptive analysis was used to analyze the epidemiological characteristics of HFMD reinfection, and spatial autocorrelation analysis to identify geographic clusters of cases. Cox proportional hazards regression was used to evaluate reinfection risk factors.

**Results:**

During the study period, 16,766 HFMD reinfection cases involving 8,210 individuals were reported in the district, with a reinfection rate of 6.41%. The interval between the two infections ranged from 0.06 to 9.92 years, and 77.42% of patients were reinfected within 2.5 years. There was a bimodal distribution in time (May-July and September-October). The spatial distribution of HFMD reinfections predominantly followed a random pattern, with cold spots clustered in a non-urban sub-district. Cox proportional hazards regression revealed significantly lower risks of HFMD reinfection among children aged >3 years, in early childhood education and care, students, and females. Individuals initially infected with prevalent strains of CV-A16, CV-A6, or other enteroviruses exhibited significantly higher risks of HFMD reinfection. Temporally, reinfection risks were significantly higher during epidemic years and peak transmission periods. Spatially, elevated HFMD reinfection risks were observed in urban areas, high population density regions, and areas with greater medical resource availability.

**Conclusion:**

HFMD reinfections in a district of southern China demonstrated distinct population heterogeneity, clustering temporally during epidemic peaks and spatially in urban centers with high population density and advanced medical resources. These findings underscore the need for a targeted early-warning system and enhanced control measures in high-risk transmission hotspots.

## 1 Introduction

Hand, foot, and mouth disease (HFMD) is an acute enteroviral infection primarily affecting children under 5 years old worldwide (1–5). It demonstrates high transmissibility and poses a substantial global public health burden, with particularly high incidence across Asia-Pacific populations (4, 6, 7). Among the key challenges posed by HFMD, reinfection stands out as a significant public health concern (8, 9). Recurrent infections may progressively elevate the risk of severe disease (10–12), creating a dual burden on both families and society (13, 14). It not only exacerbates the overall disease burden but also complicates global prevention and control strategies.

HFMD is caused by multiple enterovirus serotypes, such as EV-A71, CV-A16, CV-A6, and CV-A10 (15, 16). A critical characteristic of these serotypes is their lack of cross-protective immunity (17–21), which serves as the key driver of HFMD reinfection. Notably, reinfection is not determined by a single factor, its occurrence is further modulated by demographic characteristics, epidemic cycles, and regional heterogeneity. Individual factors such as the initially infecting viral strain and age may elevate reinfection risk by modulating host immune status (22, 23). At the temporal level, reinfection risk varies by epidemic year and season, driven by viral virulence and transmissibility. While population density potentially enhances transmission efficiency, socioeconomic factors and healthcare access mediate their effects via economic constraints, health literacy, and care utilization patterns (17). However, systematic study addressing these multifaceted determinants remains inadequate. Besides, existing studies have primarily focused on the central (24, 25), eastern (26–29), southwestern (9, 30), and northwestern China (31). Evidence from southern China, a region with distinct socioeconomic characteristics, remains scarce. Existing studies provide insufficient evidence to formulate precise control strategies for HFMD reinfection.

Located in the core of the Guangdong-Hong Kong-Macao Greater Bay Area, this district of southern China has a permanent population of 4.47 million, comparable to a mid-sized Chinese prefecture-level city, and exhibits high population density and significant mobility. This district is an endemic area for HFMD. Therefore, this study aims to systematically identify the key determinants of recurrent HFMD infections in a district of southern China, examining individual characteristics, epidemic cycles, and regional patterns. The findings will provide scientific evidence for targeted precision prevention and control of HFMD reinfection in the study region and areas with similar epidemic characteristics, while also contributing critical research evidence to global, multi-dimensional HFMD prevention and control strategies.

## 2 Materials and methods

### 2.1 Sources of information

We collected HFMD case data from the China Information System for Disease Control and Prevention (CISDCP) for patients residing in a district of southern China, with symptom onset between January 1, 2008, and June 30, 2024. The dataset included gender, age, demographic classification, residential area, severe-case status, and etiological test results.

The Center for Disease Control and Prevention (CDC) in the district and local healthcare providers conducted rigorous multi-level quality control procedures to guarantee the accuracy and consistency of infectious disease surveillance data. Healthcare facilities assign dedicated staff to collect and report infectious disease data in accordance with mandated timelines and standardized protocols.

The CDC performs standardized daily reviews of infectious disease reports from healthcare facilities. Additionally, monthly audits of outpatient and inpatient records are conducted to reduce omissions and misdiagnoses, while annual standardized training is provided to improve reporting capacity among healthcare workers.

Besides, the analysis incorporated street-level data (2008–2024) on population density, total retail sales as an economic measure, and hospital counts as a healthcare resource indicator, all sourced from official Statistical Yearbooks.

### 2.2 Definition of HFMD

Hand, foot, and mouth disease (HFMD) was diagnosed according to the 2018 diagnostic criteria issued by China's National Health Commission (WS 588–2018). A clinical case of HFMD is defined as a patient presenting with papules or vesicular rash on the hands, feet, mouth, or buttocks, with or without fever. A laboratory-confirmed case was defined as meeting any of the following criteria: detection of specific enterovirus nucleic acid sequences (e.g., CV-A16, EV-A71) in anal swab or fecal specimens; isolation and identification of HFMD-associated enteroviruses such as CV-A16 or EV-A71; presence of IgM antibodies against the relevant virus during the acute phase; or at least a fourfold increase in neutralizing antibody titers to the relevant enterovirus during the convalescent phase compared with the acute phase.

### 2.3 Definition of HFMD reinfection

A case was considered a reinfection if the interval between subsequent infections in the same patient exceeded 14 days for mild HFMD cases or 23 days for severe HFMD cases (32).

### 2.4 Inclusion and exclusion criteria

We retrieved 138,241 individual HFMD case records from CISDCP. After excluding suspected cases and duplicate reports, 136,629 cases remained. The inclusion and exclusion criteria for HFMD reinfection cases were established based on previous studies. Cases were included if they met any of the following criteria: matching ID numbers, identical parental names and birthdates, or identical contact numbers and birthdates. Based on these criteria, we identified 6,865, 12,000, and 16,972 cases, respectively. All cases identified through these criteria were deduplicated, ensuring that cases matched by multiple criteria were counted only once. The final reinfection total of 18,240 cases represents the union of the three sets and is smaller than their sum due to overlaps.

**Figure 1:**
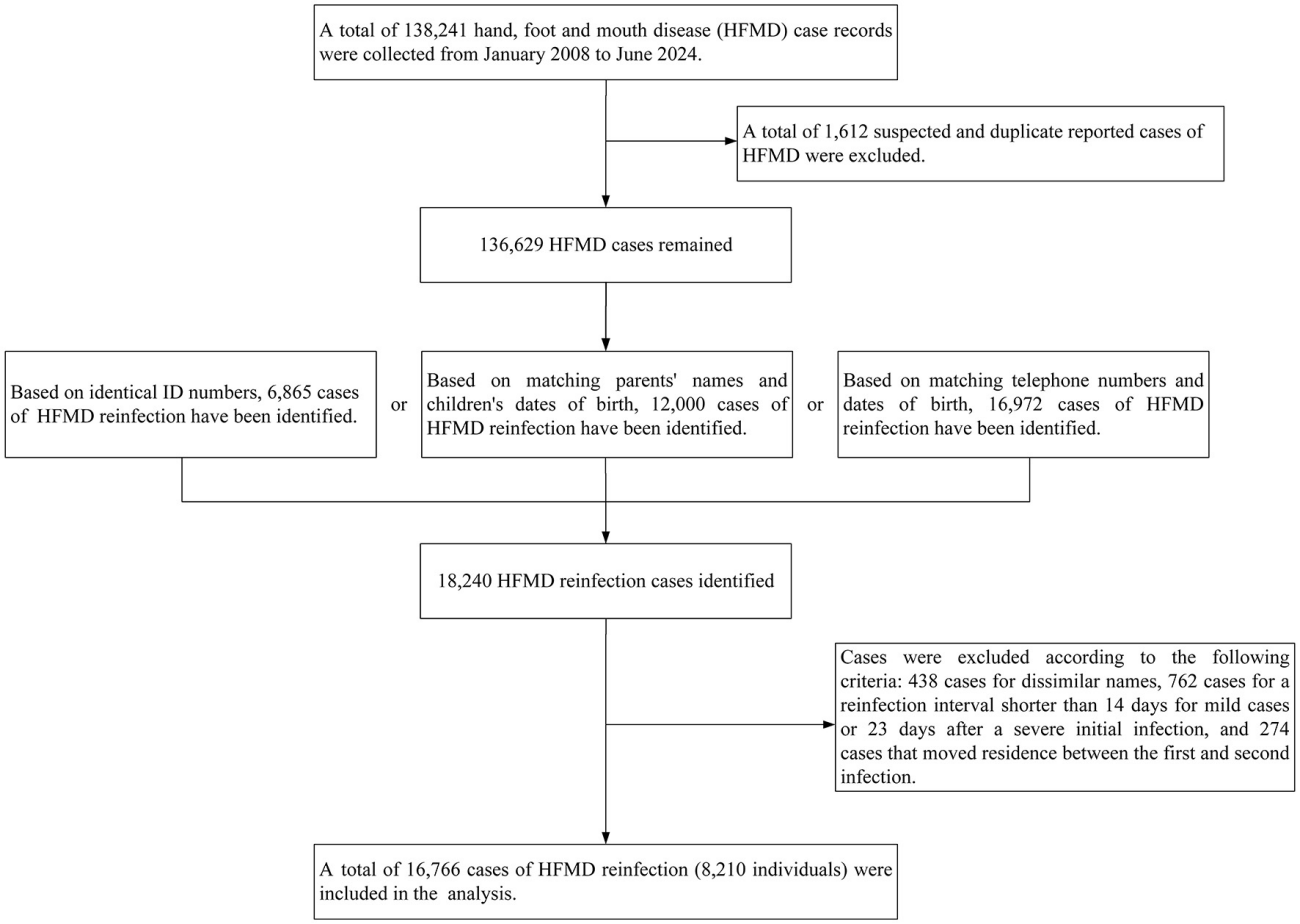
The data cleaning process for hand, foot, and mouth disease reinfection cases in a district of southern China, 2008–2024.

Cases were excluded according to the following criteria: 438 cases for dissimilar names, 762 cases for a reinfection interval shorter than 14 days for mild cases or 23 days after a severe initial infection, and 274 cases that moved residence between the first and second infection. After applying these exclusion criteria, 8,210 cases (comprising 16,766 HFMD reinfection records) remained for analysis. The process diagram of data cleaning is shown in Figure 1.

### 2.5 Statistical analysis

We used descriptive epidemiology to examine the population characteristics, temporal patterns, and geographic distribution of reinfection cases. The HFMD reinfection rate was calculated as the proportion of individuals who experienced reinfection among all individual cases of HFMD in the study.

We examined the epidemiological characteristics of reinfection cases during both primary and secondary infections. The epidemiological characteristics between HFMD reinfection cases and non-reinfection cases were compared using chi-square tests or *t*-tests where appropriate.

We analyzed temporal trends by plotting annual variations in HFMD reinfection rates over the study period. Seasonal patterns of reinfection were assessed using the seasonal index, defined as the average reinfection cases in a specific month divided by the overall monthly mean. Months with a seasonal index above 1 indicated higher reinfection activity (9). To further characterize seasonal variations, we calculated the coefficient of variation (CV) for the seasonal index as the standard deviation divided by the mean, where lower values indicated stronger seasonality (9). Additionally, we generated temporal distribution plots to visualize HFMD reinfection patterns, encompassing both symptom onset timing and intervals between consecutive infections.

For spatial analysis of HFMD reinfection cases in the district, we conducted both global and local spatial autocorrelation analyses. Global spatial autocorrelation was assessed using Moran's I index to determine whether reinfection cases exhibited spatial clustering, where values greater than 0 indicate positive spatial correlation and values less than 0 suggest negative spatial correlation. For local spatial autocorrelation, we calculated the Getis-Ord Gi^*^ index to identify statistically significant hotspots (Gi^*^>0, indicating spatial clusters of high values) and cold spots (Gi^*^ < 0, representing spatial clusters of low values).

Cox proportional hazards regression, a widely used method in survival analysis, has extensive applications in medical research. To assess risk factors for HFMD reinfection, we performed Cox proportional hazards regression analyses. Time-zero was defined as the date of onset of the first HFMD infection. An HFMD reinfection event was defined as a new clinical episode occurring ≥14 days following a mild primary infection, or ≥23 days following a severe primary infection. Participants were censored under the following rules: first, those who did not experience reinfection by the end of the observation period; second, those who died during follow-up, censored at the date of death. Person-time at risk was accumulated from the date of first HFMD onset until reinfection and censoring due to study end, or death. Total follow-up time was aggregated across all participants and reported in person-months. Cumulative risk probabilities were visualized with survival curves, while differences between subgroups were evaluated using the log-rank test. Hazard ratios (HRs) were calculated to quantify the association between exposures and reinfection risk, where an HR greater than 1 indicates increased risk, while an HR less than 1 suggests a protective effect.

To assess potential risk factors for HFMD reinfection, we analyzed various variables structured along four dimensions: demographic, clinical, temporal, and geographic. Demographic factors comprised age ( ≤ 3, >3), gender (male, female) and population classification (scattered children: young children not enrolled in formal childcare or school education; children in early childhood education and care; students; others: individuals not fitting the scattered children, children in early childhood education and care, or student categories, including teachers, healthcare workers, commercial service personnel, and etc.). Clinical factors included initial infection severity (severe, mild), predominant virus strain during the first infection year (EV-A71, CV-A16, CV-A6, Other enteroviruses), EV-A71 vaccination status, and etiological test results (EV-A71, CV-A16, EV-A71 and CV-A16, not detected). The predominant circulating strain during each participant's first infection year was determined using the district's etiological surveillance data and incorporated into the analysis as the prevalent strain for that year (Supplementary Table S1). The EV-A71 vaccine was launched in China in late 2016. To account for the potential impact of EV-A71 vaccination, we introduced a dummy variable representing population vaccination status into the model. This study assumes that EV-A71 vaccine coverage rates have increased incrementally each year since 2016. Therefore, the year variable can serve as a proxy indicator for the duration of the vaccination program and cumulative population coverage within the model. We assigned numerical values sequentially starting from 0 for 2016 (and earlier years), incrementing this value by 1 each subsequent year, reaching 6 in 2022, 7 in 2023, and 8 in 2024.

We examined the temporal effects of HFMD epidemic years and seasonal peak periods in our analysis (Supplementary Table S2). Epidemic years were characterized by an upward trend in annual reinfection rates or peak values in the district (2009, 2011, 2012, 2014, 2015, 2016, 2018, 2021, 2024), while non-epidemic years corresponded to declining trends or the lowest rates (2008, 2010, 2013, 2017, 2019, 2020, 2022, 2023), as determined from trends of HFMD reinfection rates in different years (Supplementary Figure S1). Months with a seasonal index greater than 1 are defined as peak periods (May to July, September to October), and those with a seasonal index less than 1 are defined as off-peak periods (January to April, August, November to December) (Table 2).

Furthermore, the study assessed how reinfection rates were influenced by geographic factors including residential area (urban, rural), population density (low, high), economic development levels (low, high), and medical resource (low, high) (Supplementary Table S2). Using data extracted from the District Statistical Yearbook, this study examined population density, total retail sales of consumer goods (as an economic indicator), and the number of hospitals (a proxy for medical resources) across all streets in the district. The first two were classified as high or low levels according to the median. A number of hospitals ≥2 or ≤ 1 was defined as having more or less medical resources respectively.

The data collection, processing, cleaning and analysis were conducted using R software (version 4.3.2). Spatial autocorrelation was analyzed with the “spdep” package in R. We conducted Cox proportional hazards regression analyses and generated cumulative risk probability plots using the “survival” package. Statistical significance was defined as *p* < 0.05.

## 3 Results

### 3.1 The epidemiological characteristics of reinfection cases

#### 3.1.1 Population characteristics

From January 2008 to June 2024, a total of 128,073 individual cases of HFMD were reported in the district. Among these cases, 8,210 individuals experienced recurrent infections (≥2 episodes), representing an individual-level reinfection rate of 6.41% (8,210/128,073). Among the participants, 7,886 cases (96.05%) were infected twice, 303 cases (3.69%) three times, 20 cases (0.24%) four times, and 1 case (0.02%) five times (Table 1).

**Table 1:** Epidemiological characteristics of cases of reinfection of pathogens causing hand, foot and mouth disease in a district of southern China, 2008-2024.

Characteristics	The first infection (***n*** = 8,210)		The second infection (***n*** = 8,210)		The third to fifth infections (***n*** = 324)	
	**Number of cases (** ***n*** **)**	**Proportion (%)**	**Number of cases (** ***n*** **)**	**Proportion (%)**	**Number of cases (** ***n*** **)**	**Proportion (%)**
Age (years old)						
< 0.5	139	1.69	8	0.10	0	0.00
0.5–	1,711	20.84	191	2.32	3	0.93
1–	3,399	41.40	1,645	20.04	35	10.80
2–	2,753	33.53	4,754	57.90	191	58.95
5–	202	2.46	1,530	18.64	89	27.47
≥10	6	0.08	82	1.00	6	1.85
Population classification						
Scattered children	7,205	87.76	4,705	57.31	143	44.14
Children in early childhood education and care	965	11.75	2,973	36.21	147	45.37
Students	35	0.43	529	6.44	34	10.49
Others	5	0.06	3	0.04	0	0.00
Severe case						
No	8,204	99.93	8,209	99.99	324	100.00
Yes	6	0.07	1	0.01	0	0.00
Pathogen detection results^a^						
EV–A71^b^	34	65.39	41	49.40	3	0.93
CV–A16^c^	4	7.69	15	18.07	0	0.00
EV–A71 and CV–A16	10	19.23	17	20.48	2	0.62
Others	4	7.69	10	12.05	0	0.00

^a^The number of tests for the first, second and third to fifth infections was 52 cases, 83 cases and 5 cases respectively.

^b^Enterovirus 71.

^c^Coxsackievirus group A type 16.

The epidemiological characteristics of HFMD reinfection cases are presented in Table 1. The median age of first infection was 1.56 years (range 0.03–37.98), followed by 3.38 years (0.04–38.09) for secondary infection. For cases with 3 to 5 infections, this value increased to 4.08 years (0.84–12.78). The kernel-density plot illustrated that the age at first infection was characterized by a sharply peaking curve centered at 1–2 years old, which pointed to the predominance of first infections in young infants, while the age at reinfection corresponded to a curve with a later peak (2–4 years old) and a more dispersed distribution (Supplementary Figure S2). Among the first, second, and third to fifth infection cases, scattered children represented the predominant group (87.76% for first infection, 57.31% for second infection). For cases with three to five infections, children in early childhood education and care (45.37%) and scattered children (44.14%) were the main population categories. The incidence of severe cases was 0.07% (6/8,210) during first infections and 0.01% (1/8,210) during second infections, with no severe cases observed in subsequent infections (third to fifth). All five severe cases occurred in pediatric patients.

#### 3.1.2 Time distribution

Between 2008 and 2018, the reinfection rate of HFMD exhibited a fluctuating but overall increasing trend, peaking at 10.36% in 2018. Afterward, it declined with fluctuations until 2023, followed by a rise to 10.60% in 2024 (Supplementary Figure S1). The seasonal distribution of HFMD reinfection cases displayed bimodal peaks, occurring primarily from May to July and September to October (Table 2, Supplementary Figure S3).

**Table 2:** Seasonality index and coefficients of variation of HFMD reinfection individuals in a district of southern China, 2008–2024.

Month	Reinfection	Seasonality index	Mean	Standard deviation	Coefficients of variation
1	50	0.11	5.55	5.85	1.05
2	49	0.10	4.90	3.69	0.75
3	132	0.21	10.15	11.59	1.14
4	551	0.71	34.43	49.88	1.45
5	1,596	2.05	99.75	128.79	1.29
6	1,687	2.48	120.50	130.02	1.08
7	987	1.35	65.80	65.07	0.99
8	714	0.98	47.60	45.47	0.96
9	1,136	1.46	71.00	70.89	1.00
10	837	1.15	55.80	70.62	1.27
11	317	0.41	19.81	24.23	1.22
12	154	0.23	11.00	9.33	0.85
Total	8,210	1.00	48.58	75.99	1.56

The time intervals between the onset of 8,210 reinfection cases varied from 0.06 to 9.92 years, with a mean of 1.66 years and a median of 1.08 years. The interval from first to second infection ranged from 0.06 to 9.92 years (mean: 1.67; median: 1.08). A similar pattern emerged for second-to-third infections (range: 0.06–8.01 years; mean: 1.68; median: 1.11) and third-to-fourth infections (range: 0.09–5.51 years; mean: 1.44; median: 1.02). A Kruskal–Wallis H test showed no significant difference in the distributions of these three intervals (χ^2^ = 0.678, *P* = 0.713). Only one case of fifth reinfection was observed, occurring 1.20 years after the fourth infection (Supplementary Figure S4).

#### 3.1.3 Spatial distribution

**Figure 2:**
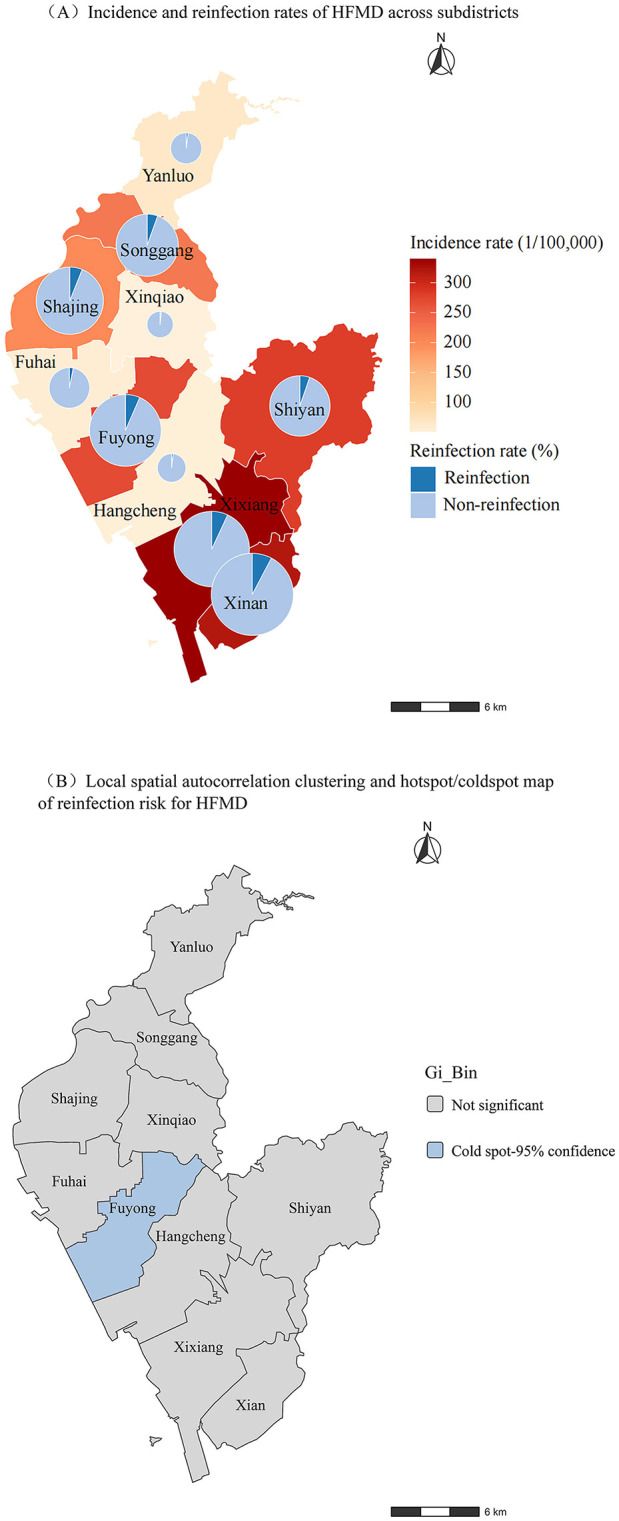
Regional distribution of HFMD reinfections across subdistricts in a southern Chinese district, 2008-2024. **(A)** Incidence and reinfection rates of HFMD across subdistricts; **(B)** Local spatial autocorrelation clustering map of reinfection risk for HFMD.

The regions with the highest number of HFMD reinfection cases were Xixiang Sub-district (2,475 cases, 30.15%) and Xin'an Sub-district (1,950 cases, 23.75%), ranking first and second, respectively (Supplementary Figure S5). The lowest number of HFMD reinfection cases occurred in Yanluo Sub-district (14 cases, 0.17%), Xinqiao Sub-district (15 cases, 0.18%), and Hangcheng Sub-district (16 cases, 0.19%) (Supplementary Figure S5). Xin'an Sub-district had the highest reinfection rate at 7.70% (1,950/25,325), followed by Xixiang Sub-district with 6.95% (2,475/35,625) (Figure 2A and Supplementary Figure S5). The lowest reinfection rates were observed in Xinqiao Sub-district (1.49%, 15/1,005), Hangcheng Sub-district (1.74%, 16/917), and Yanluo Sub-district (2.02%, 14/693) (Figure 2A and Supplementary Figure S5).

Moran's I spatial autocorrelation analysis revealed no significant spatial autocorrelation between reinfection rates and their spatial lag values (I = −0.11, *p* > 0.05). The Moran scatter plot (Supplementary Figure S6) showed data points distributed relatively evenly across all four quadrants, with a regression slope approaching zero, further supporting the absence of distinct spatial clustering or systematic spatial patterns in reinfection rates. Local spatial autocorrelation analysis revealed statistically significant cold spots (areas with lower reinfection rates) only in Fuyong Sub-district, located outside the urban core (Figure 2B). No significant spatial clustering was observed in other regions (*p* > 0.05).

#### 3.1.4 Pathogenic characteristics

The pathogenic analysis revealed that EV-A71 was the predominant pathogen in the infection cases. Specifically, it accounted for 65.39% of first infections, 49.40% of second infections, and 0.93% of third to fifth infections (Table 1). All three cases were laboratory-confirmed during both infection episodes, with EV-A71 being the most prevalent serotype (66.67%) followed by CV-A16 and other enterovirus types, which accounted for the remaining 33.33% of cases (Supplementary Table S3).

### 3.2 Analysis of influencing factors

Chi-square analysis revealed statistically significant (*p* < 0.05) associations between HFMD reinfection and multiple factors, including EV-A71 vaccination, age, population classification, circulating strain during the first infection episode, pathogen detection results, occurrence during an epidemic year, urban residence, population density, economic level, and healthcare resource (Supplementary Table S4).

Multivariate analysis demonstrated a significant trend change in HFMD reinfection following EV-A71 vaccination (*P* < 0.001) (Table 3). Individuals aged over 3 years showed a significantly lower reinfection risk compared to those aged 3 years or younger (HR = 0.48, 95% CI 0.44–0.53). Children attending early childhood education and care (HR = 0.78, 95% CI 0.72–0.84), students (HR = 0.21, 95% CI 0.15–0.29), and other grouped populations (HR = 0.13, 95% CI 0.06–0.33) all exhibited significantly lower reinfection risks compared to scattered children. Females showed a significantly lower risk of reinfection (HR = 0.81, 95% CI 0.78–0.85) compared to males. Primary infection with either CV-A16 (HR = 2.65, 95%CI: 2.14–3.29), CV-A6 (HR = 3.74, 95%CI: 3.04–4.60), or other enteroviruses (HR = 3.56, 95%CI: 2.91–4.37) was associated with a higher risk of reinfection.

**Table 3:** Cox proportional hazards regression analysis on influential factors for reinfection of pathogens causing hand, foot and mouth disease in a district of southern China, 2008–2024.

Characteristics	Single-factor analysis			Multivariate analysis		
	**HR**	**95%CI**	***P***	**HR**	**95%CI**	***P***
EV-A71 vaccination	0.83	(0.82, 0.84)	< 0.001	0.86	(0.85, 0.88)	< 0.001
Sex						
Male						
Female	0.83	(0.79, 0.86)	< 0.001	0.81	(0.78, 0.85)	< 0.001
Age (years old)						
≤ 3						
≥3	0.30	(0.28, 0.32)	< 0.001	0.48	(0.44, 0.53)	< 0.001
Population classification						
Scattered children						
Children in early childhood education and care	0.47	(0.44, 0.50)	< 0.001	0.78	(0.72, 0.84)	< 0.001
Students	0.08	(0.06, 0.11)	< 0.001	0.21	(0.15, 0.29)	< 0.001
Others	0.06	(0.02, 0.14)	< 0.001	0.13	(0.06, 0.33)	< 0.001
Severe case						
No						
Yes	0.99	(0.45, 2.19)	0.990	1.11	(0.48, 2.55)	0.814
Prevalent strain in the year of first infection						
EV–A71						
CV–A16	1.52	(1.24, 1.87)	< 0.001	2.65	(2.14, 3.29)	< 0.001
Other enteroviruses	3.78	(3.10, 4.60)	< 0.001	3.56	(2.91, 4.37)	< 0.001
CV–A6	1.96	(1.60, 2.39)	< 0.001	3.74	(3.04, 4.60)	< 0.001
Pathogen detection results						
EV–A71						
CV–A16	0.41	(0.15, 1.17)	0.095	0.79	(0.28, 2.25)	0.664
EV–A71 and CV–A16	0.71	(0.35, 1.43)	0.337	1.11	(0.55, 2.26)	0.770
Other enteroviruses	0.48	(0.17, 1.37)	0.170	0.54	(0.19, 1.54)	0.251
Not detected	1.23	(0.88, 1.72)	0.232	1.04	(0.74, 1.48)	0.809
Epidemicyear						
No						
Yes	1.38	(1.32, 1.45)	< 0.001	1.24	(1.14, 1.35)	< 0.001
Peakperiod						
No						
Yes	1.01	(0.96, 1.06)	0.700	1.07	(1.02, 1.13)	0.008
Residential area						
Rural area						
Urban area	1.30	(1.24, 1.35)	< 0.001	1.13	(1.02, 1.25)	0.022
Population density						
Low						
High	1.22	(1.16, 1.27)	< 0.001	1.30	(1.01, 1.68)	0.046
Economic level						
Low						
High	1.22	(1.17, 1.28)	< 0.001	0.82	(0.63, 1.06)	0.131
Medical resource						
Low						
High	1.34	(1.28, 1.40)	< 0.001	1.22	(1.13, 1.32)	< 0.001

HR, Hazard Ratio; CI, Confidence Interval.

Compared to non-epidemic periods, children demonstrated a significantly elevated risk of HFMD reinfection during epidemic years (HR = 1.24, 95% CI 1.14–1.35). Moreover, the reinfection risk was notably elevated during epidemic peaks (HR = 1.07, 95% CI 1.02–1.13) relative to non-peak periods (Table 3).

Urban populations (HR = 1.13, 95%CI: 1.02–1.25) exhibited a higher reinfection risk compared to non-urban residents. Areas with high population density (HR = 1.30, 95%CI: 1.01–1.68) showed a higher risk of reinfection compared to low-density regions. Individuals residing in areas with a higher number of hospitals (HR = 1.22, 95%CI: 1.13–1.32) exhibited an increased risk of reinfection (Table 3).

The results of single-factor analysis were similar to those of multi-factor analysis (Table 3). Besides, it showed that people in regions with a higher economic level had a higher risk of reinfection.

**Figure 3:**
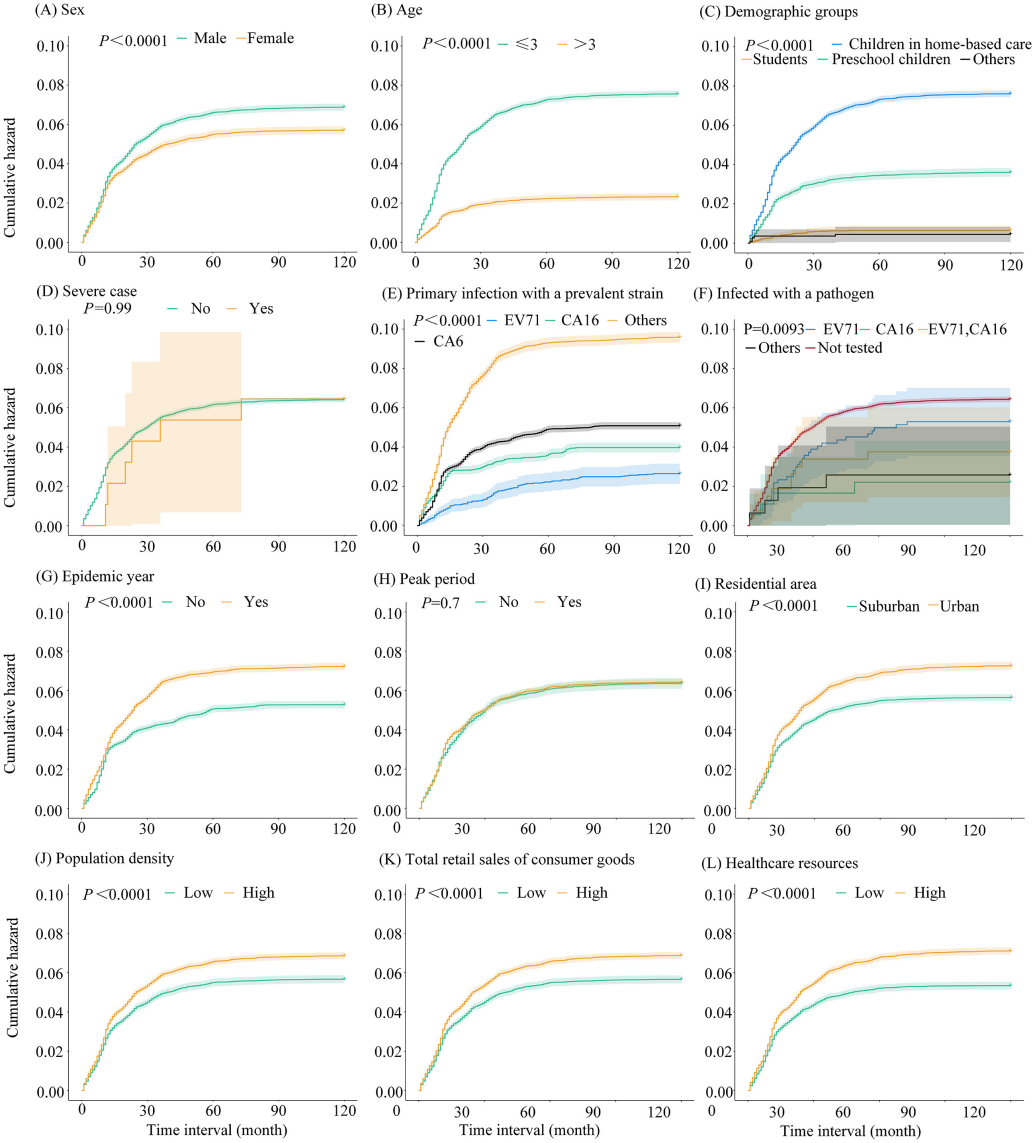
Kaplan-Meier curves for HFMD reinfection risk in the overall population and subgroups in a district of southern China, 2008–2024. **(A)** Sex; **(B)** Age; **(C)** Demographic groups; **(D)** Severe case; **(E)** Primary infection with a prevalent strain; **(F)** Infected with a pathogen; **(G)** Epidemic year; **(H)** Peak period; **(I)** Residential area; **(J)** Population density; **(K)** Total retail sales of consumer goods; **(L)** Healthcare resources.

The risk of HFMD reinfection showed a linear increase within the first 30 months following initial infection, after which the upward trend gradually plateaued. Among the 8,210 cases analyzed, 77.42% (6,356/8,210) experienced reinfection within 30 months of initial infection, while 18.37% (1,508/8,210) were reinfected between 30 and 60 months, 3.40% (279/8,210) between 60 and 90 months, and 0.82% (67/8,210) after 90 months (Figure 3).

Subgroup analyses demonstrated faster initial increases in cumulative reinfection risk during the first 30 months post-infection among male patients, children aged ≤ 3 years, scattered children, urban residents during epidemic years, populations in high-density areas, and regions with greater retail consumption or enhanced healthcare resources. This accelerated risk progression subsequently decelerated (Figure 3).

## 4 Discussion

This study systematically examined factors influencing HFMD reinfection in a district of southern China, with a comprehensive analysis spanning individual characteristics, epidemic cycles, and regional factors. From 2008 to 2024, 16,766 HFMD reinfection cases involving 8,210 individuals were reported in the district, with a reinfection rate of 6.41%. The interval between the two infections ranged from 22 to 3,621 days, and 77.42% of patients were reinfected within 2.5 years. The temporal distribution exhibited two distinct peaks occurring between May to July and September to October. HFMD reinfections showed no significant spatial positive correlation in the district, though a distinct cold spot emerged in rural Fuyong Sub-district. Multifactorial Cox regression analysis revealed elevated reinfection risks associated with epidemic years, the peak transmission period, urban settings, high population density areas, abundant healthcare resources, male sex, age ≤ 3 years, scattered children, initial non-EV-A71 enteroviral infections, and shorter durations since EV-A71 vaccination.

HFMD reinfections in our study exhibited a bimodal epidemic pattern peaks in May-July and September-October, aligning with findings from Jiulongpo, Chongqing (9). The region's subtropical monsoon climate, with persistent high humidity in autumn due to coastal sea fog (33), sustains enterovirus survival and transmission (33–36). Our results showed that the risk of reinfection significantly increased during the peak period and the epidemic year. To our knowledge, only one prior study conducted in Jiulongpo, Chongqing (9) has examined the influence of epidemic periods on HFMD reinfection risk. Two mechanisms likely operate synergistically. Increased viral circulation during epidemic periods leads to greater environmental contamination, while higher caseloads facilitate contact transmission. Together, these factors contribute to elevated reinfection risks. Notably, our finding contrasts with the Jiulongpo report (9), which observed a higher reinfection risk in non-epidemic years. This discrepancy may reflect regional differences in viral-strain predominance, vaccination coverage, or baseline immunity, underscoring the need for locally tailored surveillance. Our study therefore provides novel evidence that epidemic timing constitutes a critical and region-specific determinant of reinfection risk. Strengthened local surveillance and machine learning-based forecasting of HFMD, combined with tiered digital alerts and precision controls (37), should therefore be prioritized during identified epidemic periods optimize pre-epidemic interventions and resource allocation.

Our study demonstrated a 1.128-times higher reinfection rate in urban vs. rural areas, consistent with findings from multiple regions including Wuhan (25), Chongqing (30), Xining (31), Wuxi (26), Quzhou (28). This urban predominance may reflect higher population density and increased mobility in metropolitan settings. Notably, to our knowledge, no prior study has specifically examined the impact of population density on HFMD reinfection. We found that HFMD reinfection rates were 1.298 times higher in high-density areas compared to low-density areas, underscoring density as a key transmission driver. This supports prioritizing interventions such as intensified health education, enhanced daily screening, and regular environmental disinfection in high-density urban and congregate settings like kindergartens.

Equally novel is our examination of medical resource level as a risk factor, which has not been previously explored in HFMD reinfection. Regions with greater medical resources showed a higher reinfection risk. This phenomenon may be caused by a combination of multiple factors. While factors like health literacy disparities and risks of hospital-acquired infection are relevant (38), the core explanation for the observed pattern is surveillance bias. Regions with more abundant medical resources demonstrate more thorough case detection and reporting. Conversely, areas with limited healthcare infrastructure may harbor substantial undetected or unreported infections. Consequently, this finding reflects disparities in regional access to medical care and disease surveillance capabilities. Future work requires integrating active surveillanc data, such as community infection rate surveys, to distinguish actual risk from surveillance bias.

Consistent with previous studies (28, 29, 31), Children under 3 years old and scattered children face a higher reinfection risk. The increased susceptibility in young children may be attributed to underdeveloped immune systems, suboptimal hygiene conditions, and greater exposure to pathogens. In line with prior studies (9, 29, 30), our results indicate a significantly elevated reinfection risk among males. This observed sex disparity may reflect the synergistic contribution of both behavioral patterns and immunological differences. Behaviorally, male children typically participate more frequently in outdoor activities and have broader social contact networks (39–41), coupled with lower adherence to personal hygiene practices (42), collectively increasing their exposure risk. Immunologically, males exhibit weaker humoral responses to enteroviruses and higher fecal pathogen detection rates, suggesting inherent sex-based differences in susceptibility (43). In summary, these findings underscore the importance of targeted public health measures. These measures include focused hygiene education for families with scattered children under 3 years of age. Such education should emphasize key hygiene practices including frequent handwashing, routine cleaning and disinfection of toys and tableware, as well as surface disinfection. Additionally, sex–specific behavioral guidance is needed. For example, encouraging improved personal hygiene and reinforcing handwashing after outdoor group activities, particularly for boys, can help mitigate reinfection risk in the most vulnerable subgroups. Furthermore, consistent with prior studies, this study indicates no significant association between initial infection severity and the risk of reinfection (28, 32). A severe initial episode of HFMD neither prevents reinfection nor increases susceptibility to reinfection. This may be due to limited statistical power from the small number of severe cases, or it may suggest that children develop similar levels of immunity post-recovery, regardless of initial disease severity.

Notably, reinfection risk was significantly decreased when the initial infection year's predominant strain was EV-A71, relative to periods dominated by other serotypes. It aligns with findings from Wuhan (25), Chongqing (30), Quzhou (28) and Anhui Province (24), potentially due to variations in subtype shedding rates. Previous studies indicate that EV-A71 demonstrates slower viral shedding kinetics and prolonged clearance time compared to CV-A16 (44). The prolonged viral clearance period may lead to sustained immune activation against EV-A71, which could lower the likelihood of subsequent infections (44). The analysis further revealed a negative correlation between the duration of EV-A71 vaccination (since its implementation in 2016) and the risk of reinfection, with longer vaccination periods associated with progressively lower reinfection rates. This phenomenon effectively reflects the use of vaccination duration as an indirect measure of population coverage. As vaccination programs persist over time, the proportion of children completing the immunization series gradually accumulates within the target population. This accumulation significantly weakens the transmission intensity of EV-A71. These findings underscore the importance of maintaining and optimizing EV-A71 vaccination. Given that HFMD remains the most reported category C infectious disease in China, its disease burden continues to be substantial. However, the current EV-A71 monovalent vaccine provides no crossprotection against other major circulating serotypes such as CV-A16 and CV-A6, limiting its overall effectiveness. Thus, advancing the development of multivalent vaccines that target multiple prevalent serotypes is an urgent public-health priority for achieving broader and more sustainable HFMD control (45).

This study has several limitations that should be considered. First, the laboratory-confirmed etiological results were constrained by the available diagnostic reagents, which preferentially detected EV-A71 and only sporadically identified CV-A16, with no further typing performed for other enterovirus serotypes. The resulting heterogeneity in detection rates across cases could introduce selection bias, compounded by potential information bias arising from inter-hospital variability in testing capabilities. Second, the dataset only includes records up to June 30, 2024. Some cases initially reported in 2023 with subsequent reinfections were excluded, potentially leading to an underestimation of the true reinfection rate. Third, the extremely limited sample size (*n* = 3) of cases with both primary and repeat laboratory-confirmed infections precludes meaningful determination of whether reinfections were caused by the same or different viral strains. Fourth, Reinfection rates might be underestimated due to the lack of data on residential relocation, which prevented adjustment for migration out of the study area. Fifth, a limitation lies in the absence of individual-level data on EV-A71 vaccination status, preventing direct adjustment for this critical potential confounder. To partially address this issue, we employed calendar year as a surrogate indicator for population-level cumulative vaccination coverage, based on the assumption that coverage has shown a progressive upward trend since the vaccine's inclusion in 2016. Previous studies have indicated that climatic and environmental factors may influence enterovirus survival and transmission. Future studies should further investigate their potential role in HFMD reinfection to improve the interpretability of findings.

## 5 Conclusion

In summary, HFMD reinfection was more serious in the district of southern China from 2008 to 2024. At the individual level, boys under 3 years old, particularly those with first infections caused by non-EV-A71 enteroviruses circulating that year, along with those who received EV-A71 vaccination more recently, show higher susceptibility to reinfection. Besides, reinfection rates elevated temporally during epidemic peaks and spatially in urban centers with high population density and advanced medical resources. These findings underscore the need for a targeted early-warning system and enhanced control measures in high-risk transmission hotspots. Subsequently, further research will be conducted on the influence mechanism of climate and environment on the transmission of hand, foot, and mouth disease to provide a basis for optimizing prevention and control strategies.

## Data Availability

The original contributions presented in the study are included in the article/Supplementary material, further inquiries can be directed to the corresponding author.
