# Supplementary material for: Epidemiological characteristics and influencing factors of hand, foot and mouth disease reinfection cases in a large district of southern China
Source: Front Public Health. 2026 Apr 1;14:1773908. doi: 10.3389/fpubh.2026.1773908 (PMC13079185; doi:10.3389/fpubh.2026.1773908)
Supplement: Supplementary file 1 [file Supplementary_file_1.docx]

Supplementary materials

# Supplementary Tables and Figures

- 1. **Supplementary Tables**

**Supplementary Table 1.** The dominant strain in the year of the first infection in a district of southern China

| Year | The dominant strain |
| --- | --- |
| 2008-2011 | EV-A71 |
| 2012-2016 | Other enteroviruses |
| 2017, 2019-2023 | CV-A6 |
| 2018, 2024 | CV-A16 |

**Supplementary Table 2.** Temporal and regional-level factors and indicators

| Factor | Indicator | Unit |
| --- | --- | --- |
| Time |  |  |
| Epidemic year (Yes/No) | Reinfection rate | / |
| Peak periods (Yes/No) | Seasonal index | / |
| Space |  |  |
| Urban area (Yes/No) | / | / |
| Population | Population density | People per square kilometer |
| Economic level | Total Retail Sales of Consumer Goods | Ten thousand Yuan |
| Medical resources | The number of hospitals | / |

**Supplementary Table 3.** Etiology of the first infection and the second infection of

pathogens causing HFMD in a district of southern China, 2008−2024

| First infection | The second infection | Number of cases |
| --- | --- | --- |
| EV-A71 | EV-A71 | 2 |
| EV-A71 | CV-A16 | 0 |
| EV-A71 | EV-A71 and CV-A16 | 0 |
| EV-A71 | Others | 0 |
| CV-A16 | EV-A71 | 0 |
| CV-A16 | CV-A16 | 0 |
| CV-A16 | EV-A71 and CV-A16 | 0 |
| CV-A16 | Others | 1 |
| EV-A71 and CV-A16 | EV-A71 | 0 |
| EV-A71 and CV-A16 | CV-A16 | 0 |
| EV-A71 and CV-A16 | EV-A71 and CV-A16 | 0 |
| EV-A71 and CV-A16 | Others | 0 |
| Others | EV-A71 | 0 |
| Others | CV-A16 | 0 |
| Others | EV-A71 and CV-A16 | 0 |
| Others | Others | 0 |

**Supplementary Table 4.** Analysis of Characteristics of Reinfected and Non-reinfected Patients with HFMD

| Characteristics | Number of cases（%） | Reinfecitons | Non-reinfections | Reinfeciton rate  （%） | χ^2^（P）  /T（*P*） |
| --- | --- | --- | --- | --- | --- |
|  |  | N（%）/Mean(SD) | N（%）/Mean(SD) |  |  |
| EV-A71 vaccination | 128073 | 1.36(±2.05) | 2.47（±2.73） | - | 46.262（＜0.01） |
| Sex |  |  |  |  | 70.267（＜0.01） |
| male | 76884 | 5289 | 71595 | 6.88 |  |
| female | 51189 | 2921 | 48268 | 5.71 |  |
| Age (years old) |  |  |  |  |  |
| ≤3 | 112129 | 8002 | 104127 | 7.14 | 790.360（＜0.01） |
| ＞3 | 15944 | 208 | 15736 | 1.30 |  |
| Population classification |  |  |  |  |  |
| Scattered children | 94754 | 7205 | 87549 | 7.60 | 943.370（＜0.01） |
| children in early childhood education and care | 26773 | 965 | 25808 | 3.60 |  |
| Students | 5428 | 35 | 5393 | 0.64 |  |
| Others | 1118 | 5 | 1113 | 0.45 |  |
| Severe case |  |  |  |  |  |
| No | 127980 | 8204 | 119776 | 6.41 | 1.8212e-27（1） |
| Yes | 93 | 6 | 87 | 6.45 |  |
| Prevalent strain in the year of first infection |  |  |  |  |  |
| EV-A71 | 3783 | 100 | 3683 | 2.64 | 1254.10（＜0.01） |
| CV-A16 | 23640 | 939 | 22701 | 3.97 |  |
| Other enteroviruses | 45598 | 4373 | 41225 | 9.59 |  |
| CVA6 | 55052 | 2798 | 52254 | 5.08 |  |
| Pathogen detection results |  |  |  |  |  |
| EV-A71 | 642 | 34 | 608 | 5.30 | 13.659（＜0.01） |
| CV-A16 | 181 | 4 | 177 | 2.21 |  |
| EV-A71 and CV-A16 | 266 | 10 | 256 | 3.76 |  |
| Other enteroviruses | 155 | 4 | 151 | 2.58 |  |
| Not detected | 126829 | 8158 | 118671 | 6.43 |  |
| Epidemicyear |  |  |  |  |  |
| No | 53385 | 2816 | 50569 | 5.27 | 196.42（＜0.01） |
| Yes | 74688 | 5394 | 69294 | 7.22 |  |
| Peakperiod |  |  |  |  |  |
| No | 30892 | 1967 | 28925 | 6.37 | 0.117（0.733） |
| Yes | 97181 | 6243 | 90938 | 6.42 |  |
| Residential area |  |  |  |  |  |
| Non-urban area | 67125 | 3785 | 63340 | 5.64 | 139.730（＜0.01） |
| Urban area | 60948 | 4425 | 56523 | 7.26 |  |
| Population density |  |  |  |  |  |
| Low | 48216 | 2732 | 45484 | 5.67 | 71.193（＜0.01） |
| High | 79857 | 5478 | 74379 | 6.86 |  |
| Economic level |  |  |  |  |  |
| Low | 48827 | 2763 | 46064 | 5.66 | 74.110（＜0.01） |
| High | 79246 | 5447 | 73799 | 6.87 |  |
| Medical resource |  |  |  |  |  |
| Low | 50491 | 2696 | 47795 | 5.34 | 159.02（＜0.01） |
| High | 77582 | 5514 | 72068 | 7.11 |  |

- 1. **Supplementary Figures**





**Supplementary Figure 1.** HFMD reinfection rate in a district of southern China, 2008-2024.


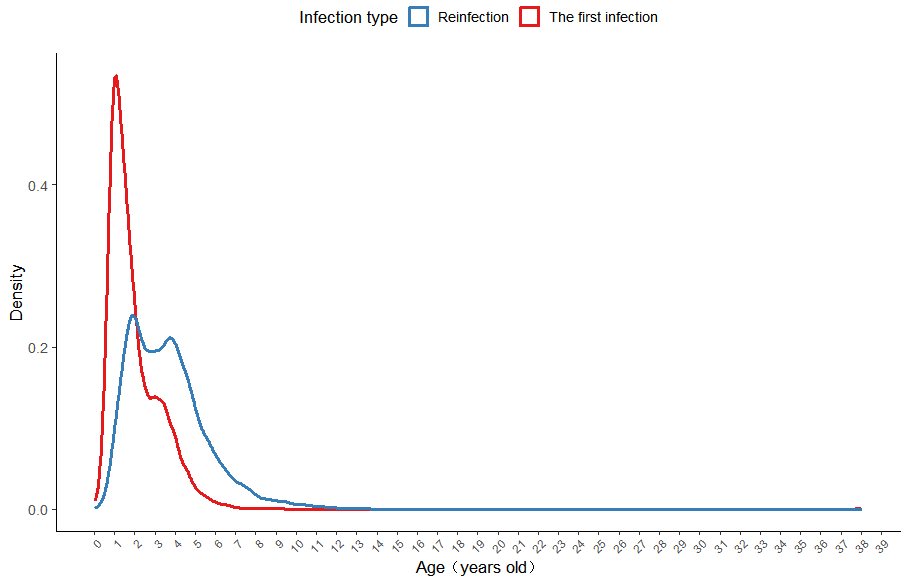


**Supplementary Figure 2.** Kernel-Density Distribution of Age at First Infection and Age at Reinfection.





**Supplementary Figure 3.** Seasonal distributions of the reinfection and non-reinfection of HFMD in a district of southern China, 2008−2024.





**Supplementary Figure 4.** Distribution of infection intervals of reinfections in a district of southern China, 2008−2024. Note: T is the interval between the onset of all reinfections. T_2-1_ is the interval between the second and first infections. T_3-2_ is the interval between the third infection and the second infection. T_4-3_ is the interval between the fourth and third infections. T_5-4_ is the interval between the fifth and fourth infections.





**Supplementary Figure 5.** The number of reinfection cases of HFMD in 10 sub-districts of the district, from 2008 to 2024.


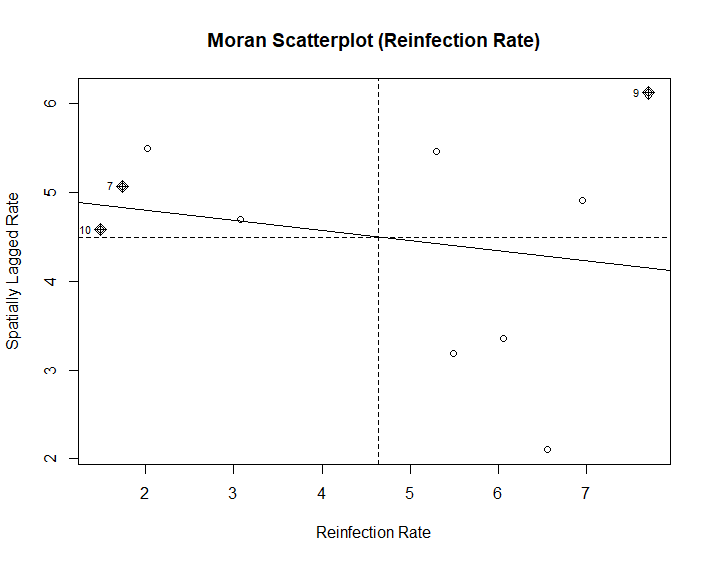


**Supplementary Figure 6.** The Moran scatter plot.
